# Root anatomy governs bi-directional resource transfer in mycorrhizal symbiosis

**DOI:** 10.1038/s41467-025-64553-1

**Published:** 2025-09-30

**Authors:** Jingjing Cao, Junjian Wang, Qingpei Yang, Binglin Guo, Tino Colombi, Oscar J. Valverde-Barrantes, Junxiang Ding, Yue Zhang, Huifang Wu, Zhipei Feng, Xitian Yang, Deliang Kong

**Affiliations:** 1 College of Forestry, Henan Agricultural University, Zhengzhou, China; 2 State Key Laboratory of Soil Pollution Control and Safety, Southern University of Science and Technology, Shenzhen, China; 3 State Environmental Protection Key Laboratory of Integrated Surface Water-Groundwater Pollution Control, School of Environmental Science and Engineering, Southern University of Science and Technology, Shenzhen, China; 4 Guangdong Provincial Key Laboratory of Soil and Groundwater Pollution Control, School of Environmental Science and Engineering, Southern University of Science and Technology, Shenzhen, China; 5 School of Biosciences, University of Nottingham, Sutton, Bonington UK; 6 Department of Soil and Environment, Swedish University of Agricultural Sciences, Uppsala, Sweden; 7 International Center for Tropical Biodiversity, Department of Biological Sciences, Florida International University, Miami, FL USA; 8 College of Ecology and Environment, Zhengzhou University, Zhengzhou, China

**Keywords:** Fungal ecology, Microbial ecology, Plant physiology

## Abstract

Plants form mycorrhizal symbioses to enhance nutrient acquisition, yet the biophysical principles governing carbon and nutrient exchange remain unclear. Here, we develop a theory of bi-directional carbon–nutrient transfer that integrates root anatomy, energetic costs, and mycorrhizal positioning. We show that nutrient uptake per unit carbon or energy investment declines with increasing root diameter due to higher carbon demands across thicker cortical tissues. Mycorrhizal fungi mitigate this constraint by enabling more carbon-efficient nutrient uptake, particularly when arbuscules are positioned in inner cortical layers. This spatial optimization minimizes the carbon cost of transporting nutrients to the stele. Our framework reconciles anatomical variation, symbiotic structure, and functional efficiency across root types and mycorrhizal strategies and offers a new lens for understanding the coevolution between roots and mycorrhizal fungi.

## Abstract (web summary)

This study develops a biophysical theory explaining root anatomical allometry as a regulator for carbon-nutrient exchange with mycorrhizal fungi. Accordingly, arbuscules residing inner cortical layers can improve carbon efficiency in nutrient uptake.

## Root anatomical allometry and the mycorrhizal symbiosis

**Fig. 1:**
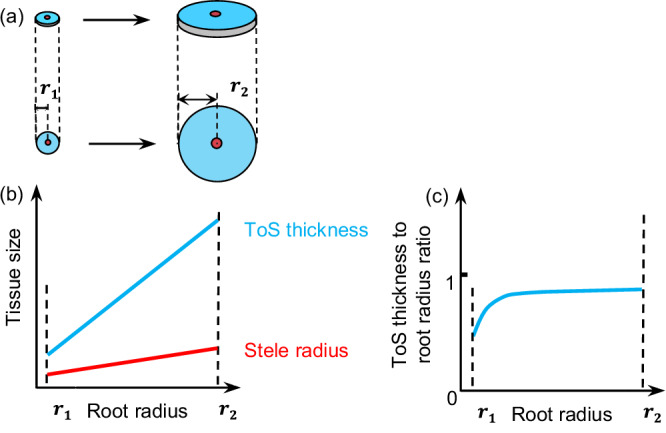
Conceptual diagram illustrating root anatomical allometry. Along with the shift of absorptive root radius from ${r}_{1}$ to ${r}_{2}$ (**a**), ToS thickness (the solid blue line) increases much steeper than stele radius (the solid red line) does (**b**), and the ToS thickness progressively dominates root radius (**c**). This is called allometric assembly of root anatomical structures, i.e., root anatomical allometry. Root cross-sectional area, modified from Figure 1 in our previous study^19^, corresponding to roots with radius ${r}_{1}$ and ${r}_{2}$ is shown above the allometric lines. Stele: the area within the red circle. ToS (the tissues outside the stele): the area between the blue and red circles.

Root nutrient uptake is essential for plant growth and ecosystem functioning^1–4^ and is undertaken by a few terminal root branches that typically lack secondarily-developed tissue^5,6^. These ‘absorptive roots’ consist of two concentric cylindrical structures with distinct functions: first, tissues outside the stele (ToS, including epidermis, exodermis, and cortex) responsible for nutrient uptake and symbiotic associations with mycorrhizal fungi^7,8^, and second, the stele responsible for nutrient, water, and carbon transport^5,9^. Mounting evidence highlights that ToS thickness increases much more steeply than stele radius with increasing root radius (*r*) across plant species, and a phenomenon referred to as ‘root anatomical allometry’ (Fig. 1a,b). Evidently, ToS thickness progressively dominates root radius along with increasing root radius (Fig. 1c). Like *Archimedes’ fulcrum to move the world*, this globally occurring allometric relationship^7,10–14^ is fundamental for our understanding of the vast diversity in root form and function^3,8,11,15,16^, plant evolution and plant responses to global change^10,17,18^.

Recent theoretical and empirical studies highlight a critical role of mycorrhizal associations in shaping root anatomical allometry^19,20^. Most terrestrial plants have symbiosis with mycorrhizal fungi, which exchange soil-derived nutrients for plant-supplied carbon, mostly in the form of lipids^21–24^. Despite remarkable advancements in our understanding of root-fungus interactions^21,24–29^, the role of root anatomical structures in regulating the bi-directional transfer of carbon and nutrients between roots and mycorrhizal fungi remains poorly explored. Here, we bridge root anatomical allometry with the *bi-directional carbon-nutrient transfer* in mycorrhizal plants, thereby proposing a novel functioning role of mycorrhizal fungi.

## Bi-directional carbon-nutrient transfer theory

**Fig. 2:**
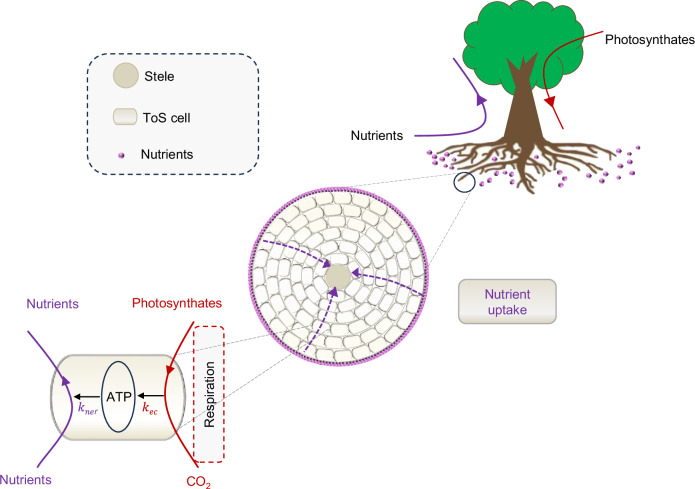
Schematic illustration of nutrient (solid purple circles) uptake by plant roots without mycorrhizal association. Cells in the ToS use energy derived from respiration to drive nutrient uptake across ToS cells (represented by dashed arrows across the ToS). ToS tissues outside the stele, ATP adenosine triphosphate, *k*_*ec*_ the amount of ATP per unit of photosynthetic carbon respired, *k*_*ner*_ the amount of nutrient uptake per unit of ATP invested by roots.

**Fig. 3:**
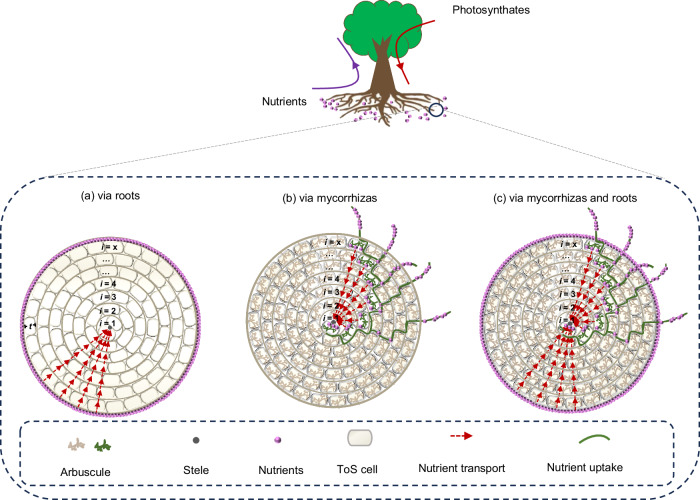
Comparative illustration of active nutrient uptake via roots only, mycorrhizas only, and roots and mycorrhizas combined. In each scenario, nutrients are actively transported to the stele through ToS cells, as indicated by dashed arrows. Arbuscules are assumed to be formedevenly across ToS cells. Root cross-sectional area is illustrated with *x* ToS cell layers. Nutrients released from the arbuscules into the root ToS cells are actively transported to the steleas indicated by the dashed arrows (**b**, **c**), similar to the case of non-mycorrhizal roots (**a**). ToS tissues outside the stele, t cell layer thickness, x number of the ToS celllayer with the innermost layer defined as layer 1, i a given ToS cell layer.

Physiologically, most soil nutrients taken up by plant roots and mycorrhizal fungi are actively transported through the symplast of the ToS, which consumes root respiration-derived adenosine triphosphate (ATP)^30–33^ (Fig. 2). Our theory is based on three key metrics: the energy yield of respiration per unit of respired photosynthetic carbon (${k}_{{ec}}$ [µmol (ATP) µmol (C)^−1^]), the nutrient (Nt) benefit of per unit energy investment to roots (${k}_{{ner}}$ [µmol (Nt) µmol (ATP)^−1^]) (Fig. 2) and the nutrient (Nt) benefit per unit carbon investment to symbiotic mycorrhizal fungi (${k}_{{ncf}}$ [µmol (Nt) µmol (C)^−1^]) (Fig. 3).

## Bi-directional resource transfer for non-mycorrhizal roots

We first outline the *bi-directional carbon-nutrient transfer theory* for the case of roots lacking mycorrhizal association (i.e., Eqs. 1–3). This baseline allows us to assess how variation in nutrient benefit per unit energy (carbon) investment in roots themselves (i.e., ${k}_{{ner}}$) with root radius compares with that in symbiotic mycorrhizal fungi (i.e., ${k}_{{ncf}}$). Comparing ${k}_{{ner}}$ and ${k}_{{ncf}}$, along with their components (i.e., nutrient benefit and carbon or energy investment), can offer fresh insights into the functions of mycorrhizal fungi beyond their traditionally recognized role in expanding the nutrient foraging area.

Consider an absorptive root with radius $r$, composed of *x* layers of ToS cells, and the innermost layer corresponding to the 1st layer. Each layer has a thickness $t$, such that $r$ can be approximated as *r* ≈ ${tx}$ (Fig. 3a)^20^, since the ToS thickness progressively becomes the dominating component of root radius with increasing *r* according to the root anatomical allometry (Fig. 1c).

For the case of no mycorrhizal association, the amount of nutrients reaching the root surface (${N}_{{ru}}$) is proportional to the root perimeter and thus the root radius (Eq. 1, Box 1): Since most of the root cross-sectional area constitutes ToS^5,7^, the energy provided by ToS cell respiration, and thus the amount of ATP generated^34^, is approximately proportional to root cross-sectional area, or to ${r}^{2}$ (Fig. 2). If we assume fully active and thus symplastic transport of nutrients reaching the root surface with no mycorrhizal symbiosis, the energy investment rate (${E}_{{{\rm{rt}}}}$) needed to enable active nutrient transport through the ToS, is approximately proportional to ${r}^{2}$ (Eq. 2, Box 1). Given that energy investment (i.e., ${E}_{{{\rm{rt}}}}$, Eq. 2) increases much faster than nutrient acquisition (i.e., ${N}_{{ru}}$, Eq. 1) with increasing root radius (Fig. 3a), the nutrient acquisition per unit energy investment for non-mycorrhizal roots (${k}_{{ner}}$) inevitably decreases with increasing root radius (Eq. 3, Box 1). Obviously, such pattern of ${k}_{{ner}}$ is attributed to greater ATP investment for active nutrient transport across the thicker ToS. Therefore, thicker absorptive roots are inherently less energy-efficient in acquiring nutrients, revealing a key biophysical constraint on root function in the absence of mycorrhizal symbiosis.

Box 1: Mathematical description of the bi-directional carbon-nutrient transfer theory **Without mycorrhizal colonization** (Fig. 3a)

The amount of nutrients reaching the root surface (${N}_{{ru}}$) can be approximately expressed as:

1 $${N}_{{ru}}=2\pi {rk}=2\pi {txk}$$

where ${k}$ is the rate of nutrients reaching the root per unit of root perimeter.

The energy investment rate (${E}_{{{\rm{rt}}}}$) needed for active nutrient uptake through the ToS in the absence of mycorrhizal symbiosis is given by:

2 $${E}_{{{\rm{rt}}}}={N}_{{{\rm{ru}}}}{E}_{0}{tx}=2{{\rm{\pi }}}k{E}_{0}{({tx})}^{2}$$

where ${E}_{0}$ is the energy cost of active uptake of one unit of nutrient per unit radial length across the ToS.

Nutrient acquisition per unit of energy investment of roots without mycorrhizal colonization (${k}_{{ner}}$) is then:

3 $${k}_{{ner}}=\frac{{N}_{{ru}}}{{E}_{{rt}}}=\frac{1}{{E}_{0}r}$$

**With mycorrhizal colonization** (Fig. 3b, c)

The amount of nutrient uptake via mycorrhizal fungi (${N}_{{fu}}$) is given by:

4 $${N}_{{fu}}={\sum }_{i=1}^{x}\left(2\pi {ti}{k}^{{\prime} {\prime} }\right)\,=\pi t{k}^{{\prime} {\prime} }({x}^{2}+x)$$

where ${k}^{{\prime} {\prime} }$ is the rate of nutrient uptake per unit perimeter of the ToS layer by mycorrhizal fungi in a ToS layer.

Carbon investment required for nutrient uptake via mycorrhizal fungi $({C}_{{fu}})$, including fungal biomass construction and respiratory costs, is calculated as:

5 $${C}_{{fu}}=\frac{{N}_{{fu}}}{{k}_{{ncf}}}=\,\frac{\pi t{k}^{{\prime} {\prime} }({x}^{2}+x)}{{k}_{{ncf}}}$$

where ${k}_{{ncf}}$ denotes the amount of nutrients plants receive from arbuscules (or ectomycorrhizal fungi) in exchange for one unit of carbon from roots.

The energy cost for uptake of nutrients released from AM arbuscules to the stele (${E}_{{ft}}$) is given by:

6 $${E}_{{ft}}={\sum }_{i=1}^{x}\left(2\pi {ti}{k}^{{\prime} {\prime} }t{E}_{0}\left(i-1\right)\right)=\,2\pi {t}^{2}{k}^{{\prime} {\prime} }{E}_{0}\frac{{x}^{3}-x}{3}$$

Nutrient benefit per unit energy investment for roots with mycorrhizal colonization (${{k}^{{\prime} }}_{{ner}}$) is then:

7 $${{k}^{{\prime} }}_{{ner}}=\frac{{N}_{{ru}}+{N}_{{fu}}}{{E}_{{rt}}+{E}_{{ft}}}\,=\frac{2k+{k}^{{\prime} {\prime} }\left(x+1\right)}{2{E}_{0}t\left({kx}+{k}^{{\prime} {\prime} }\frac{{x}^{2}-1}{3}\right)}$$

The energy savings of nutrient transport via mycorrhizal hyphae across a distance of $(x-i)t$, i.e., from the epidermis to the ${i}^{{th}}$ ToS layer fully occupied with arbuscules, relative to nutrient transport via root plasmodesmata (${E}_{{si}}$), can be calculated as follows:

8 $${E}_{{si}}=2\pi {tik}{\prime} {\prime} \left(x-i\right)t\left({E}_{0}-{E}_{0f}\right)=2\pi {t}^{2}k{\prime} {\prime} \left({E}_{0}-{E}_{0f}\right)\left({xi}-{i}^{2}\right)$$

The cumulative energy savings of nutrient transport via mycorrhizal hyphae for an absorptive root with $x$ ToS layers fully occupied with arbuscules (${E}_{s}$, Fig. 3a, b) can be calculated as:

9 $${E}_{s}={\sum }_{i=1}^{x}{E}_{{si}}=2\pi {t}^{2}k{\prime} {\prime} \left({E}_{0}-{E}_{0f}\right){\sum }_{i=1}^{x}\left({xi}-{i}^{2}\right)$$

Nutrient benefit per unit energy investment for uptake of the nutrients released from arbuscules in the ${i}^{{\mathrm{th}}}$ ToS layer to the stele (${k}_{{ne}{r}_{i}}$) is calculated as:

10 $${k}_{{ne}{r}_{i}}=\frac{2\pi {tik}{\prime} {\prime} }{2\pi {tik}{\prime} {\prime} {E}_{0}t(i-1)}=\frac{1}{{E}_{0}t(i-1)}$$

▓

## Bi-directional resource transfer for mycorrhizal roots

We then extend the *bi-directional carbon-nutrient transfer theory* to roots colonized by mycorrhizal fungi, treating resource flows through the fungi and the roots separately (Fig. 3b, c). For the fungi in the mycorrhizal symbiosis, nutrients are acquired by the extraradical mycelium (Eq. 4 in Box 1), while the carbon fuelling mycelium growth and respiration is supplied by host roots (Eq. 5 in Box 1) (Fig. 3b). For the roots in the mycorrhizal symbiosis, nutrient input includes two sources: directly acquired by roots themselves (Eq. 1), and transferred from mycorrhizal fungi into root ToS cells (Eq. 4). The total energy cost for the roots involves actively transporting both sources of nutrients across ToS layers (Eqs. 2 and 6; Fig. 3c). We outline the bi-directional resource transfer in mycorrhizal roots in the following steps:

First, we calculate the amount of nutrient uptake via mycorrhizal fungi, i.e., ${N}_{{fu}}$. The bi-directional transfer of carbon and nutrients between absorptive roots and mycorrhizal fungi happens through arbuscules (Fig. 3b). Following a previous study^19^, we assume an even distribution of mycorrhizal fungi within the ToS, and for simplicity, one arbuscule per unit perimeter of the ToS layer (Fig. 3b). Then, ${N}_{{fu}}$ is calculated by summing up nutrients that all root ToS layers receive from individual arbuscules (Eq. 4). It is easy to learn that ${N}_{{fu}}$ is approximately proportional to ${r}^{2}$, suggesting more nutrient uptake via mycorrhizal fungi in thicker absorptive roots.

Second, we calculate the carbon investment required for nutrient uptake via mycorrhizal fungi, i.e., ${C}_{{fu}}$, including fungal biomass construction and respiratory costs. Nutrient uptake by mycorrhizal fungi is undertaken by extraradical mycelium and then transported to intraradical structures of roots (Fig. 3b). While calculating ${C}_{{fu}}$ based on extraradical traits (e.g., hyphal length, soil nutrient availability, and the proportion of nutrients transferred to roots from extraradical mycelium^26^) would require complex and variable-dependent modeling, we simplify the approach by focusing on the intraradical mycelium and ignore detailed biochemical processes of active nutrient transport by enzymes (usually described by Michaelis-Menten equation). From this perspective, the amount of carbon allocation from roots to mycorrhizal fungi (to maintain the growth and metabolism of the extraradical and intraradical mycelium) is proportional to the amount of nutrients transferred from fungi to roots; that is, ${C}_{{fu}}$ is proportional to ${N}_{{fu}}$. Since ${N}_{{fu}}$ scales with root cross-sectional area, ${C}_{{fu}}$ is approximately proportional to ${r}^{2}$ (Eq. 5), suggesting more carbon investment for nutrient uptake via mycorrhizal fungi in thicker absorptive roots.

Third, we calculate the energy investment for active transport of nutrients released from the arbuscules to the stele, i.e., ${E}_{{ft}}$. For simplicity, the nutrients released from arbuscules in the innermost ToS layer ($i$ = 1, Fig. 3b) are assumed to move directly into the stele. Nutrients released from arbuscules in the 2nd ToS layer ($i$ = 2, Fig. 3b) need to move across one ToS layer corresponding to a distance of $t$ before reaching the stele. Therefore, nutrients released from arbuscules in the *i* ToS layer need to move across $i-1$ ToS layers before reaching the stele. Calculating the energy needed for active transport of the arbuscule-released nutrients in each ToS layer to the stele, we can get ${E}_{{ft}}$, which is approximately proportional to ${r}^{3}$ (Eq. 6 in Box 1). Note that ${E}_{{ft}}$ is attributed to roots, as root respiration provides the energy needed for such active transport.

**Fig. 4:**
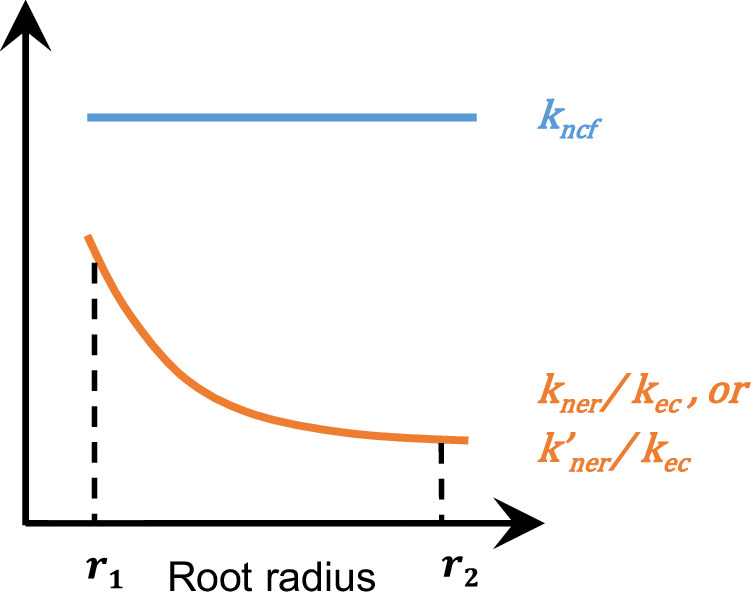
Different patterns of nutrient benefit per unit carbon investment to roots (${k}_{ner}/{k}_{ec}\,{{\rm{or}}}\,{k}_{ner}^{{\prime} }/{k}_{ec}$) and mycorrhizal fungi (*k*_*ncf*_) as a function of absorptive root radius ranging from *r*_1_ to *r*_2_. See Eqs. 3 and 7 and the main text for how the nutrient benefit per unit carbon investment to absorptive roots or mycorrhizal fungi varies with root radius (*r*). ${k}_{{ner}}$ nutrient benefit per unit energy investment to roots non-mycorrhizal roots, ${{k}^{{\prime} }}_{{ner}}$ same for mycorrhizal roots, *k*_*ec*_ amount of ATP per unit of photosynthetic carbon respired.

Finally, we calculate the nutrient benefit per unit energy investment for roots with mycorrhizal colonization, i.e., ${{k}^{{\prime} }}_{{ner}}$. Evidently, the total nutrient benefit in mycorrhizal roots includes both root-derived (i.e., ${N}_{{ru}}$) and arbuscule-derived nutrients (i.e., ${N}_{{fu}}$). Meanwhile, the total energy investment comprises ${E}_{{{\rm{rt}}}}$ and ${E}_{{ft}}$. Therefore, we can see that ${{k}^{{\prime} }}_{{ner}}$, similar to the non-mycorrhizal case (i.e., ${k}_{{ner}}$ in Eq. 3), also decreases with increasing root radius (Eq. 7 in Box 1; Fig. 4). This suggests that even in the presence of mycorrhizal symbiosis, thicker absorptive roots themselves are also less energy-efficient in nutrient acquisition.

## Implications

### Contrasting patterns of *k*_*ner*_ and *k*_*ncf*_ with root radius

Our analysis of bi-directional carbon-nutrient transfer in both mycorrhizal and non-mycorrhizal roots reveals contrasting patterns in ${k}_{{ner}}$ and ${k}_{{ncf}}$ with increasing root radius (Fig. 4). Specifically, for absorptive roots, the nutrient benefit per unit carbon investment decreases with increasing root radius, irrespective of their mycorrhizal associations (${{k}^{{\prime} }}_{{ner}}$/${k}_{{ec}}$, Eq. 7) or not (${k}_{{ner}}$/${k}_{{ec}}$, Eq. 3). However, for mycorrhizal fungi, the nutrient benefit per unit carbon investment, i.e., ${k}_{{ncf}}$, is likely independent of root radius. This is because the bi-directional exchange between root-derived carbon and arbuscule-released nutrients is governed by a suite of transporters (e.g., SRT and STR2) and transcriptional factors (e.g., PHR, Pho4, WRI5a/CBX1, and RAM1) that function similarly across ToS cell layers with mycorrhizal association^21,28,35–38^. Therefore, ${k}_{{ncf}}$ of mycorrhizal fungi in different ToS layers could be relatively constant. Similarly, mycorrhizal roots of different diameters (i.e., different numbers of ToS layers) can have similar ${k}_{{ncf}}$ of mycorrhizal fungi when mycorrhizal composition is the same. It is well-known that certain AM fungal species provide more nutrients per unit carbon supply from host roots than other AM species^27,39–42^. For EM species, short-distance hyphal exploration types usually have higher nutrient return per unit carbon investment, i.e., higher ${k}_{{ncf}}$ of the mycorrhizal fungi^43^, relative to long-distance hyphal exploration types. Therefore, ${k}_{{ncf}}$ of mycorrhizal community within the roots, compared with ${k}_{{ner}}$/${k}_{{ec}}$, or ${{k}^{{\prime} }}_{{ner}}$/${k}_{{ec}}$ of the roots, is less affected by root radius but more likely affected by mycorrhizal composition (Fig. 4).

The advantage of mycorrhizal fungi in ${k}_{{ncf}}$ is attributed to two key properties of intraradical and extraradical hyphae. Property 1: intraradical mycorrhizal hyphae, especially those with arbuscules occurring in the middle to inner ToS layers, can effectively reduce the distance and energy cost of active transport of nutrients across the ToS to the stele (Fig. 3b, c). This is because nutrients acquired from these hyphal tubes (2–30 μm in diameter) can bypass the narrow and high-resistance plasmodesmata (40–50 nm in diameter) of the outer root cells^23,44^, effectively acting as a low-resistance “highway” for radial nutrient movement. Property 2: mycorrhizal hyphae are much thinner than plant roots, which, in contrast to roots, can result in much lower energy costs for nutrient transport from hyphae surface to inner hyphal tubes. This could also explain the frequent observation of higher nutrient benefit per unit carbon investment for mycorrhizal fungi (${k}_{{ncf}}$) than for roots (${k}_{{ner}}$/${k}_{{ec}}$)^39,45^ (Fig. 4).

### Carbon savings of nutrient transport via mycorrhizal fungi

Given these advantages, mycorrhizal symbiosis could reduce the carbon cost of nutrient acquisition, particularly in thicker absorptive roots. Assuming that all nutrients are acquired by mycorrhizal fungi (Fig. 3b) and that the energy costs for transporting one unit of nutrient per unit radial length across the ToS via mycorrhizal hyphae is ${E}_{0f}$, the energy saving of nutrient transport via mycorrhizal hyphae can be derived in four steps:

First, compared to the high-resistance transport through root plasmodesmata (${E}_{0}$, the energy cost of active transport per unit nutrient per unit radial length across the ToS, also see Eq. 2 in Box 1), the energy savings per unit nutrient transport per unit radial length via low-resistance mycorrhizal hyphae (Fig. 3b) would be ${E}_{0}-{E}_{0f}$.

Second, for arbuscules located in the *i*th ToS layer (Fig. 3b), the transport distance from the epidermis to this ToS layer is $(x-i)t$, where *x* is the total number of ToS layers and *t* is the thickness of each layer. Therefore, arbuscules occupying inner ToS cells (smaller $i$) confer greater energy savings, as nutrients are transported farther through low-resistance hyphae (Fig. 3b).

Third, as aforementioned, nutrient acquisition for arbuscules fully occupying the ${i}^{{th}}$ ToS layer is $2\pi {tik}\hbox{''}$ (Eq. 4). Compared with transport through plasmodesmata, the energy savings of transporting this amount of nutrient via mycorrhizal hyphae across a distance of $(x-i)t$, i.e., ${E}_{{si}}$, can be calculated by Eq. 8 in Box 1. Hence, the energy saving of a single ToS layer (the ${i}^{{th}}$ layer), i.e., ${E}_{{si}}$, can be maximized when arbuscules occupy the center ToS layers.

Finally, summing up ${E}_{{si}}$ across all ToS layers (i.e., ${E}_{s}$ in Eq. 9 in Box 1), we can get the energy savings of nutrient transport via mycorrhizal hyphae for an absorptive root with $x$ ToS layers that are fully occupied with arbuscules (Fig. 3a, b). Accordingly, fully distributing arbuscules across a greater number of ToS layers (larger *x*) could yield higher overall energy savings, i.e., a larger ${E}_{s}$.

### Optimized positioning of the resource transfer within roots

Our theory offers novel insights into the spatial distribution of the carbon-nutrient bi-directional transfer between roots and mycorrhizal fungi within the roots. To illustrate this, we define another term, ${k}_{{ne}{r}_{i}}$, that depicts nutrient benefit per unit energy investment for active transport of the arbuscule-derived nutrients from the $i$th ToS layer to the stele (Fig. 3b, c; Eq. 10 in Box 1).

Although more outer ToS layers have more arbuscule-derived nutrients (approximately proportional to $r$), more energy investment from roots (approximately proportional to ${r}^{2}$) is needed for active nutrient transport from the arbuscules to the root stele. Therefore, ${k}_{{ne}{r}_{i}}$ tends to increase from outer to inner ToS layers. In contrast to ${k}_{{ne}{r}_{i}}$, nutrient benefit per unit carbon investment into mycorrhizal fungi, i.e., ${k}_{{ncf}}$, is less affected by the number of ToS layers. Therefore, positioning arbuscules in the inner or middle ToS layers (i.e., a smaller *i*; Fig. 3b, c) can enhance energy savings of nutrient transport via both roots (Eq. 10) and mycorrhizal fungi (see the previous section). The energy savings could be greater in thicker absorptive roots (larger *x*) when the arbuscules reside deeper within the cortex (Fig. 3b, c).

**Fig. 5:**
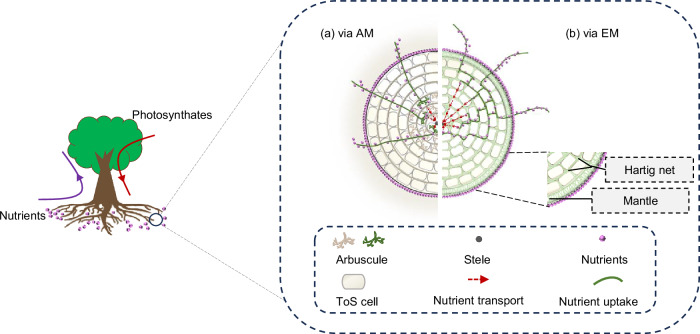
Nutrient uptake via arbuscular mycorrhizas (AM) only or via ectomycorrhizas (EM) only in extant scenario of mycorrhizal association. Arbuscules in AM roots are formed in the inner instead of outer ToS layers (**a**) and EM hyphae also tend to release nutrients to the inner ToS layers. Rootcross-sectional area is illustrated with seven ToS cell layers as in Fig. 3. EM hyphal mantle and Hartig net are illustrated in **b**. ToS tissues outside the stele.

Empirical evidence supports this optimization: mycorrhizal colonization is often concentrated in the middle and inner ToS layers^22,46–48^, and we interpret this as the extant scenario of mycorrhizal association (Fig. 5a). This mycorrhizal positioning likely enhances energy utilization efficiency for plants forming mycorrhizal symbioses, whether obligately (always forming associations) or facultatively (forming them only in some environments). Occasionally, mycorrhizal colonization can suppress root nutrient uptake by down-regulating genes encoding nutrient transporters in root epidermis^49^. Under such antagonistic conditions^49^, plants may rely more heavily on mycorrhizal fungi for nutrient acquisition. Locating more arbuscules in the middle and inner ToS layers could be beneficial because, as aforementioned, it reduces the energetic cost of moving nutrients from cortical cells to the stele.

Although we propose the new theory using AM roots as an example, this theory may also apply to other mycorrhizal types (e.g., EM roots). In EM roots, the bi-directional transfer of carbon and nutrients between roots and EM fungi occurs in root tissues where the Hartig net (Fig. 5b) is formed^50^. Similar to AM roots, the majority of the bi-directional resource transfer in EM roots might occur in the middle and inner ToS layers connecting with the Hartig net, thereby optimizing nutrient benefit per unit energy investment to the EM roots. Mycorrhizal colonization usually decreases for plants in fertile soils. Consequently, for plant species growing in nutrient-poor soils, we expect that the bi-directional resource transfer between roots and mycorrhizal fungi occurs preferentially in more inner root cortical layers, as this reduces the energy cost of active transport of the mycorrhizal fungi-released nutrients to the stele.

### Root anatomical allometry and the resource transfer

In the root anatomical allometry, ToS thickness increases much more steeply than the stele radius with increasing root radius (Fig. 1a, b). Therefore, ToS thickness comprises a progressively larger proportion of root radius and cross-sectional area in thicker absorptive roots (Fig. 1c). In other words, this anatomical allometry ensures disproportionately thicker ToS in thicker absorptive roots.

This allometric assembly of root anatomical structures has important implications for the energetic efficiency of resource exchange. In thicker absorptive roots, the dominance of thickened ToS means greater energy investment is required for active nutrient transport across these tissues, leading to a lower nutrient benefit per unit energy investment (${k}_{{ner}}$). However, when arbuscules are located in the inner ToS layers of thicker roots, the energy cost for arbuscule-released nutrient transport to the stele is reduced. Together, root anatomical allometry provides a structural basis for the energy-efficient, bi-directional resource exchange between roots and mycorrhizal fungi.

### Implications of the bi-directional resource transfer theory

The *bi-directional carbon-nutrient transfer theory* outlined here provides a novel perspective on the symbiotic relationship between plant roots and AM fungi. Traditionally, this symbiosis has been considered to be formed to greatly enlarge nutrient foraging area because mycorrhizal hyphae are much thinner than roots^25,26,51,52^. This is especially the case for AM plants with relatively thick absorptive roots, which are well-known to depend more on mycorrhizal fungi for nutrient acquisition than plants with thinner absorptive roots^24,53^. As outlined in our new theory, this greater dependence on mycorrhizal fungi in thicker AM roots could result from a decrease of ${k}_{{ner}}$ in thicker absorptive roots while ${k}_{{ncf}}$ remains relatively constant (Fig. 4).

The new theory could represent a novel understanding of the coevolution of roots and mycorrhizal fungi under environmental changes. Plant roots have evolved to be thinner with less mycorrhizal associations since the mid-Cretaceous, when plants experienced physiological drought due to a decline in atmospheric CO_2_ concentration (from about 1130 ppm^54^)^15,55–57^. Generally, plants may enhance mycorrhizal dependence under the CO_2_ declining-induced carbon limitation condition because carbon investment per unit nutrient benefit is higher for roots (${k}_{{ec}}$/${k}_{{ner}}$) than for mycorrhizal fungi (1/${k}_{{ncf}}$) (Fig. 4). However, the CO_2_ declining-induced root thinning, as aforementioned (Eqs. 3 and 7), can cause a disproportional increase of ${k}_{{ner}}$ of the roots (Fig. 4), and hence reduce mycorrhizal dependence in thinner absorptive roots.

Last but not least, our finding that mycorrhizal association serves as a carbon-saving strategy (i.e., reducing carbon cost per unit nutrient acquisition, especially in thicker absorptive roots, also see Eq. 9) does not contradict the long-held view that carbon (energy) is rarely a constraint for plants, especially for trees^51,58^). While carbon is abundant in many plant tissues, root growth and maintenance rely heavily on current photosynthate^59^. Consequently, the “saved” carbon through more efficient nutrient uptake by mycorrhizal fungi in thicker absorptive roots could be repurposed for supporting more extensive extraradical mycelial networks, enhancing root activity, stimulating rhizosphere exudation, or building carbon reserves to improve stress resilience^60^.

### Concluding remarks and future perspectives

Here, by linking root anatomical allometry, a common way for root structure assembly, with the *bi-directional carbon-nutrient transfer* in mycorrhizal roots, we present a novel understanding of the function, ecology, and evolution of mycorrhizal symbiosis with roots. Future studies could improve and test our theory in several ways. For example, we can test whether the carbon or energy cost of nutrient uptake through the cortex increases with root diameter across plant species. In addition, we assumed a constant amount of nutrient uptake per unit of invested ATP for different nutrients (Fig. 2), but this amount could differ among nutrients (e.g., nitrogen vs phosphorus) and even the same nutrient in different chemical forms^32^. We also expect the preference for bi-directional resource transfer between roots and mycorrhizal fungi in the middle and inner root ToS layers. This prediction could be examined by anatomically mapping the spatial distribution of arbuscules, or by detecting expression patterns of genes involved in bi-directional resource transfer between roots and mycorrhizal fungi across cortical layers, using techniques such as single-cell RNA sequencing (scRNA-Seq)^35,36,61^. On the other hand, while some studies have shown that mycorrhizal colonization reduces carbon costs of nutrient acquisition^39,45^, empirical studies are still lacking regarding whether such carbon cost reduction is greater when fungi colonization occurs closer to the root stele. Testing these predictions in different mycorrhizal types and ecosystems will provide insight into the broader applicability of this new theory.

In addition, we assume a consistent carbon-nutrient bi-directional transfer coefficient, i.e., ${k}_{{ncf}}$, which usually occurs in controlled systems with a single mycorrhizal fungi species. However, plant roots usually harbour many mycorrhizal fungal species with different ${k}_{{ncf}}$. Therefore, one unit carbon investment to different mycorrhizal fungal communities could lead to different nutrient rewards, which could weaken the tight coupling of bi-directional resource transfer between roots and mycorrhizal fungi. Therefore, an integrated ${k}_{{ncf}}$ could be calculated by summing the product of ${k}_{{ncf}}$ and relative abundance of each mycorrhizal fungal species^27^, derived from high-throughput sequencing data. A similar way could also be used to calculate an integrated ${k}_{{ner}}$ that accounts for the diversity of nutrient forms and their concentrations.
